# A comprehensive review on pharmacological applications and drug-induced toxicity of valproic acid

**DOI:** 10.1016/j.jsps.2022.12.001

**Published:** 2022-12-09

**Authors:** Ayesha Safdar, Fatima Ismail

**Affiliations:** Department of Biochemistry, Institute of Biochemistry, Biotechnology and Bioinformatics, The Islamia University of Bahawalpur, Pakistan

**Keywords:** Valproic acid, Anticonvulsant, Mood regulator, Anticancer activity, Valproic acid toxicity

## Abstract

Valproic acid, a branching short chain fatty acid, is a popular drug to treat epilepsy and acts as a mood-stabilizing drug. The obstruction of ion channels and Gamma Amino Butyrate transamino butyrate GABA has been linked to antiepileptic effects. Valproic acid has been characterized as a Histone deacetylase inhibitor, functioning directly transcription of gene levels by blocking the deacetylation of histones and increasing the accessibility of transcription sites. Study has been extensively focused on pharmaceutical activity of valproic acid through various pharmacodynamics activity from absorption, distribution and excretion particularly in patients who are resistant to or intolerant of lithium or carbamazepine, as well as those with mixed mania or rapid cycling.

## Graphical abstract

**Figure:**
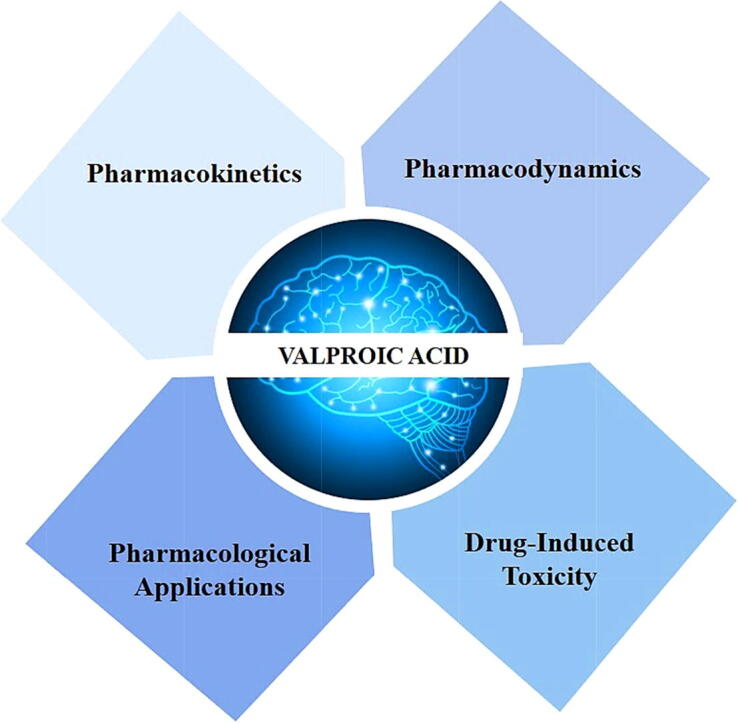

## 1 Introduction

**Fig. 1:**
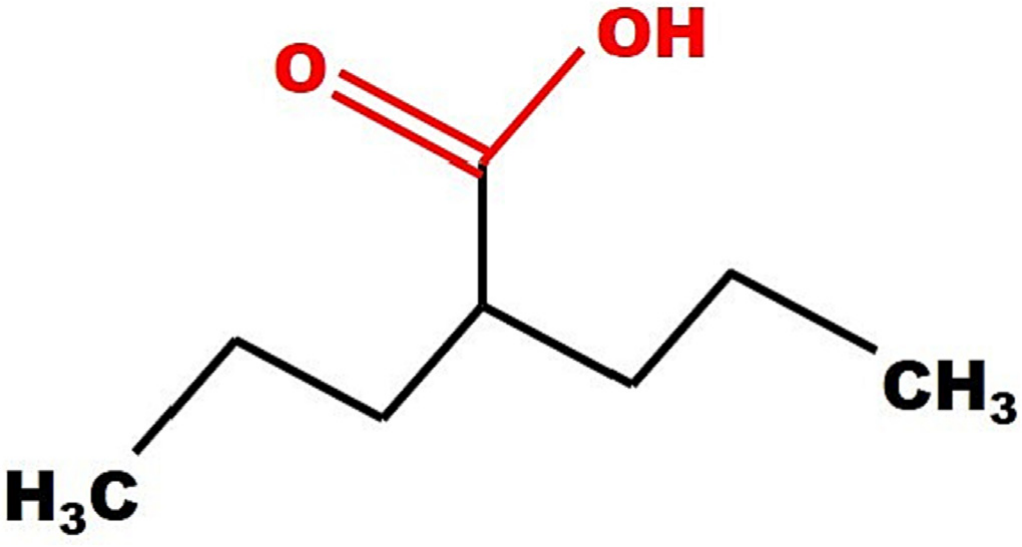
Valproic acid structure (Adewole et al., 2021).

Valproic acid (VPA), often known as valproate a fatty acid derivative and an anticonvulsant (López-Muñoz et al., 2012). For nearly a century, it was a prominent organic solvent in industry and pharmaceutical manufacture (Beckner, 1979). Antiepileptic drug (AED), Valproic acid (2-*n*-propylpentanoic acid) is traditionally utilized to treat particular seizures and epilepsy (Chou et al., 2008). Valproic acid activity is mediated by a rise in glutamatergic and γ-aminobutyric acid (GABA) Fig.1 neurotransmission (Braconnier et al., 2018). VPA is also used as a single therapy or as part of a multi-AED treatment regimen. AEDs commonly used in polytherapy with VPA include topiramate, carbamazepine, lamotrigine, and phenytoin (LaBuzetta et al., 2010). In mental health settings, valproic acid can be used and sometimes in combination with several other antipsychotic drugs to treat bipolar patients suffering from mania (Chopra et al., 2012).Fig. 1

Outbursts of aggressive behaviors in youngsters having ADHD (attention deficit hyperactivity disorder), dystonia, and some problems which impede reasoning, understanding, or learning, are also treated with VPA (Chou et al., 2008). Migraine headaches can also be treated or prevented using VPA (Mezghani et al., 2011). It was, however, unsuccessful in preventing childhood migraines (Nguyen et al., 2006). Recently, valproic acid has been utilized to cure several tumors, either alone or in conjunction with other anti-tumor medicines. It has also been proven to have neuroprotective properties in patients suffering from Alzheimer's disease. The minimal confirmation on the application of valproic acid throughout schizophrenia does not support or deny a beneficial impact (Kasiviswanathan et al., 2009). Epilepsy has a substantial cost impact both on healthcare systems and individuals. When compared to control individuals, epileptic patients had a higher annual health plan cost. This increased annual cost can be correlated with the price of AEDs, and several additional aspects of both the disease as well as associated co-morbidities (Finsterer and Zarrouk Mahjoub, 2012). Despite the fact that VPA is a relatively older AED, it continues to be a major medication because of its demonstrated remedial effects and affordable price. VPA is effective in polymedicated older epileptic patients, showing positive security records and an astonishing drug to drug interaction probability. VPA is currently available in generic form, which is less expensive than the brand form. With respect to seizure management, drug formulations are typically bioequivalent (Verrotti et al., 2011). Although the generic form can be utilized in new patients, it is not suggested for those who have efficient seizure control (Yan et al., 2021).

VPA use might be restricted due to loss or gradual decline of efficacy, as well as undesirable medication responses. The National Institutes of Health warns the patients using valproic acid about the danger of hazardous liver and pancreas harm associated with this drug's use. Anorexia, vomiting, nausea, and eventually somnolence are the most prevalent initial signs of toxicity caused by valproic acid, which is frequently supported by enhanced convulsions. Coma, Jaundice, and coagulation problems, are all possibilities. Hypoglycemia and ascites are possible. A fairly typical sign of liver failure is coma, which has been developing over time. Drowsiness, headache, dizziness, diarrhea, heartburn, constipation, alterations in one's appetite, back pain, weight changes, abnormal thinking, mood swings, agitation, memory loss, loss of coordination, uncontrollable shaking, blurred or double vision, uncontrollable movements of the eyes, ringing in the ears, sore throat, hair loss, and stuffed or runny nose are some other ADRs related to Valproic acid usage. ADRs associated with severe VPA include spots of purple color on the skin, unusual bruising or bleeding, blisters or rash, fever, itching, confusion, hives, enlarged glands, trouble breathing or swallowing, joint weakness, suicidal thoughts, and depression (Gayam et al., 2018). VPA has been studied for its anti-migraine, neuroprotective, and anti-manic properties. Recently, it is a chemical of attention in the field of oncology due to its properties that are anti-proliferative, it is also the subject of a lot of clinical trials for different kinds of cancer.

## 2 Pharmacokinetics

VPA has a poor clearance of 6–20 ml/h/kg due to its high protein binding (87–95 %) (Leppik and Birnbaum, 2010). In humans, there are three routes of Valproic acid metabolism: glucuronidation, mitochondrial oxidation (both considered significant channels, accounting for about 50 and 40 percent of the dosage, respectively), as well as cytochrome P450 mediated oxidation (a peripheral path, showing 10 percent) (Tan et al., 2010). The main urinary byproduct of Valproic acid (30–50 %) is valproate glucuronide (Argikar and Remmel, 2009). Valproic acid is glucuronidated by UGT1A3 (UDP Glucuronosyltransferase Family 1 Member A3), UGT1A4 (UDP Glucuronosyltransferase Family 1 Member A4), UGT1A6 (UDP Glucuronosyltransferase Family 1 Member A6), UGT1A8 (UDP Glucuronosyltransferase Family 1 Member A8), UGT1A9 (UDP Glucuronosyltransferase Family 1 Member A9), UGT1A10 (UDP Glucuronosyltransferase Family 1 Member A610), UGT2B7 (UDP Glucuronosyltransferase Family 2 Member B7), and UGT2B15 (UDP Glucuronosyltransferase Family 2 Member B15), as per an in-vitro analysis of human purified recombinant proteins and liver microsomes (Chung et al., 2008). Another research has questioned UGT2B15 role, claiming that valproic acid blocks UGT2B15, however, VPA does not glucuronidated it. In vitro, UGT1A1 has no action against VPA (Ethell et al., 2003).

**Fig. 2:**
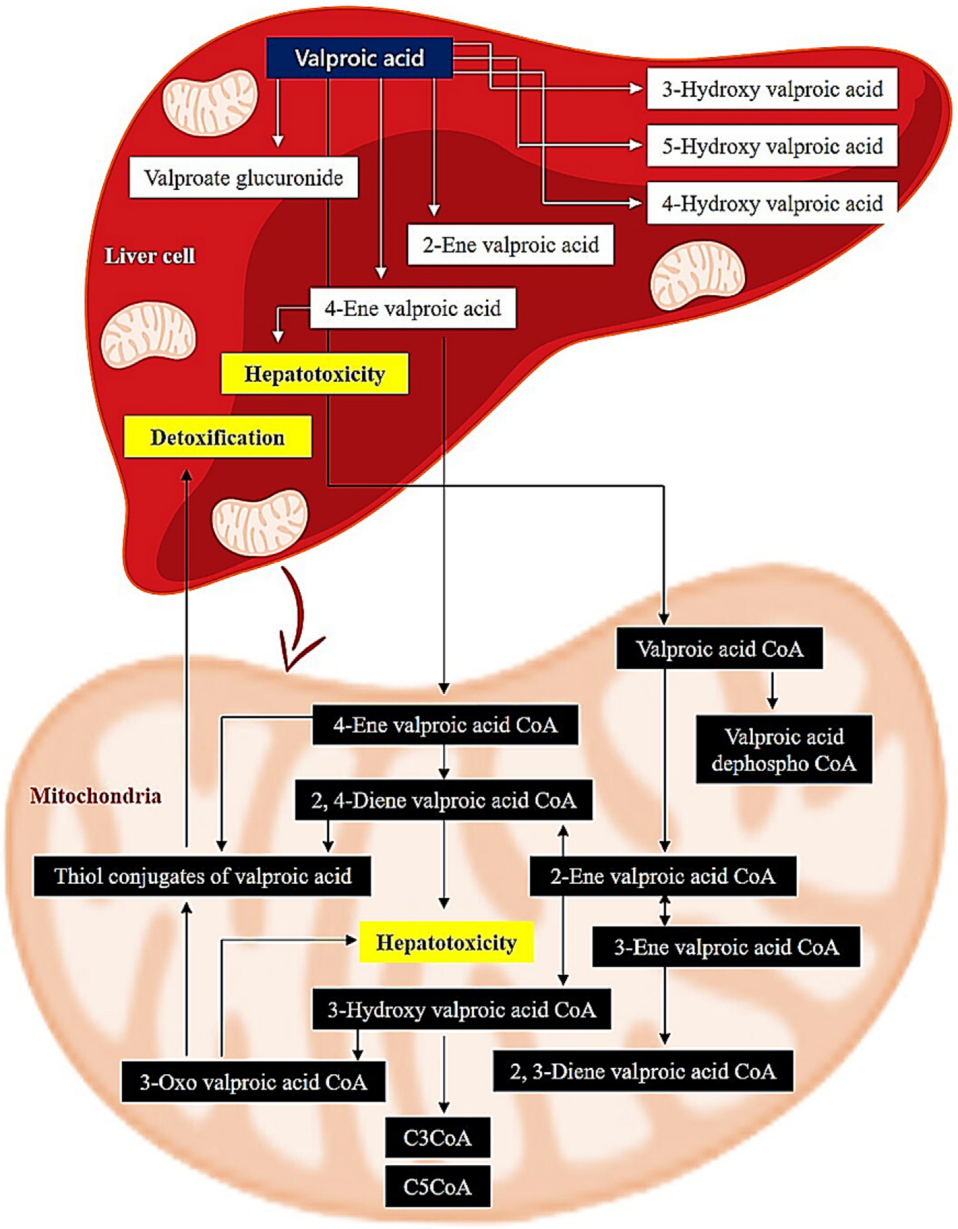
A graphical display of pharmacokinetic pathways of valproic acid (VPA) (Ghodke-Puranik et al., 2013). Completely interactive pathway may be found online at PharmGKB at http://www.pharmgkb.org/pathway/PA165964265. CYP (cytochrome P450).

VPA, a fatty acid can be processed in the mitochondria via endogenous mechanisms (Fig. 2). A few of the mitochondrial intermediates of valproic acid produced by this route have been found to be hepatotoxic. The basic interpretation of VPA bioactivation is that 4-ene-valproic acid enters into mitochondria, forming a 4-eneVPA-CoA ester with the help of ACADSB, which then undergoes β-oxidation and generates active 2,4-diene-valproic acid-CoA ester (Kassahun et al., 1994). Fluorinated analogs of 4-ene-valproic acid have been shown to inhibit β-oxidation, which prevents the fluoro analog of 4-ene-valproic acid from producing a CoA ester (Grillo et al., 2001), demonstrating that β-oxidation of 4-eneVPA plays a unique function in producing a 2,4-diene metabolite. 2,4-diene-valproic acid-S-CoA, a harmful metabolite is then combined with glutathione and produces thiol conjugates. These metabolites produced by 4-ene-valproic acid are chemically active, with the ability to diminish the mitochondrial glutathione reserves in order to produce CoA conjugates, blocking enzymes involved in the -oxidation pathway. The fact that *N*-acetylcysteine conjugates of (E)-2,4-diene-valproic acid were found in human urine showed that (E)-2,4-diene-VPA biotransformation in humans is a major source of reactive thiol conjugates of valproic acid (Gopaul et al., 2000). Intermediate 4-ene-valproic acid synthesis by CYP2A6 and CYP2C9, as well as to a smaller level by CYP2B6 is the most important group of Valproic acid pathways that are controlled by CYP. These metabolizing enzymes also play a role in the conversion of valproic acid into inert 4-OH-valproic acid and 5-OH-valproic acid. CYP2A6 also contributes to the production of 3-OH-valproic acid (Kiang et al., 2006).

## 3 Pharmacodynamics

**Fig. 3:**
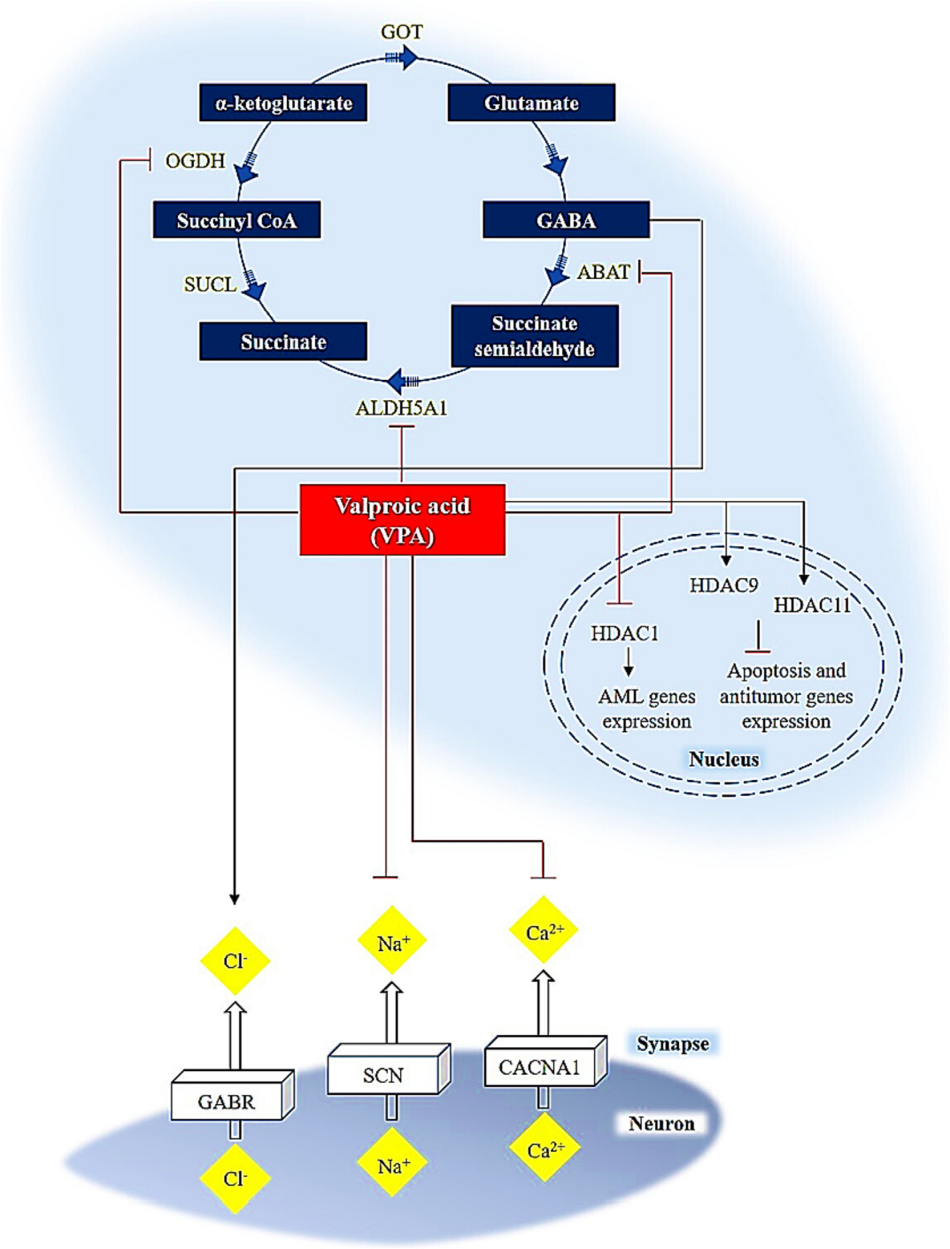
A graphical display of the potential pharmacodynamics of VPA. (Ghodke-Puranik et al., 2013). This pathway is completely interactive and may be found online at PharmGKB at http://www.pharmgkb.org/pathway/PA165959313.

VPA causes Parkinson's disease by affecting levels of GABA in the brain, which stops voltage-gated ion channels from working, and inhibiting histone deacetylases (Fig. 3). Convulsions can be caused by a loss of GABAergic inhibitory action; hence antiepileptic medicines are targeting this pathway. The tricarboxylic acid cycle produces GABA, GABA transaminase (ABAT) converts semialdehyde to succinate. Succinate semialdehyde dehydrogenase then converts succinate to succinate (ALDH5A1). OGDH (α-Ketoglutarate dehydrogenase) can turn α-ketoglutarate into succinyl CoA, which keeps it from being used to make GABA. in-vitro and ex-vivo studies have shown that valproic acid works by blocking ABAT and ALDH5A1, which are both involved in the pathway for breaking down GABA. One investigation of in-vitro type, also found that OGDH was blocked by high amounts of VPA (Johannessen and Johannessen, 2003).

VPA, in addition to raising GABA levels, may have an antiepileptic effect via lowering the high-frequency firing of neurons by closing calcium, sodium, and potassium voltage-gated channels (along with those that were coded by CACNA1C, CACNA1N, CACNA1D, also the SCN and CACNA1F family of genes) (Van den Berg et al., 1993). Valproic acid either enhances or reduces, potassium channel conductance is still debated (Chateauvieux et al., 2010). VPA has recently been discovered to be a blocker of HDAC1 and other HDACs (Phiel et al., 2001), which may boost the levels of expression of genes that are involved in apoptosis and antitumor activity. As a result, valproic acid has been presented as a potent anticancer agent. In cancer cell lines, VPA activates HDAC9 and HDAC11. Activation or overexpression of certain deacetylases in tumor cells by HDAC inhibitors can improve the effectiveness of antitumor therapy by making tumor cells die more selectively (Bradbury et al., 2005).

### 3.1 Absorption, Half-life, elimination route

The Tmax of the orally given delayed-release tablet version is four hours. Variations in the rate of absorption are thought to be from various mixtures, but this is not thought to be therapeutically significant for long-term therapy beyond affecting how often a drug is taken. Variations in absorption can result in early Tmax or elevated values of Cmax at the start of treatment, but meals can change these values in different ways. When extended-release tablet formulation was taken with food, Tmax increased from 4 to 8 h. In contrast, the sprinkle form capsules increased Tmax between 3.3 and 4.8 h. The bioavailability of all orally taken dosage forms, including those with an enteric-coating, is estimated to be over 90 %. Low-dose protein interaction appears linear, showing a free percentage of about 10 at 40 mcg/mL, but high-dose protein binding seems to be nonlinear, with a free percentage of 18.5 at 135 mcg/mL. This might be because albumin proteins have different high and low-affinity binding sites. In the aged and people with hepatic impairment, binding is likely to diminish (Georgoff et al., 2018).

VPA has a half-life of 13 to 18 h in humans (Hommers, 2020). With a pKa of 4.56, valproate, the carboxylate moiety of Valproic acid, is highly ionized, at physiological pH 7.42. Since only the lipid-soluble and non-ionized, part of VPA propagates across membranes, only a limited amount of VPA is transported into tissues via passive diffusion (Zobdeh et al., 2021). There is 70–94 % albumin-bound VPA in serum, having lesser proportions bound in women who are pregnant, older individuals, as well as in the presence of rising levels of free fatty acids. In humans, the distribution volume of valproic acid is roughly 0.14 L/kg, thus showing valproic acid is predominantly limited to bloodstream and ECFs (extracellular fluids), mostly due to ionization at normal pH and with a lot of Valproic acids linked to serum proteins. About 30–50 percent of the medication is removed by hepatic metabolism. The other main contributing route is β-oxidation in mitochondrial, accounting for approximately 40 % of the total. An additional 15 to 20 % comes from other oxidation processes. Fewer than 3 % of the total waste is excreted in urine unaltered (Baumgartner and Elger, 2020).

### 3.2 Mechanism of action and actions on the CNS

**Fig. 4:**
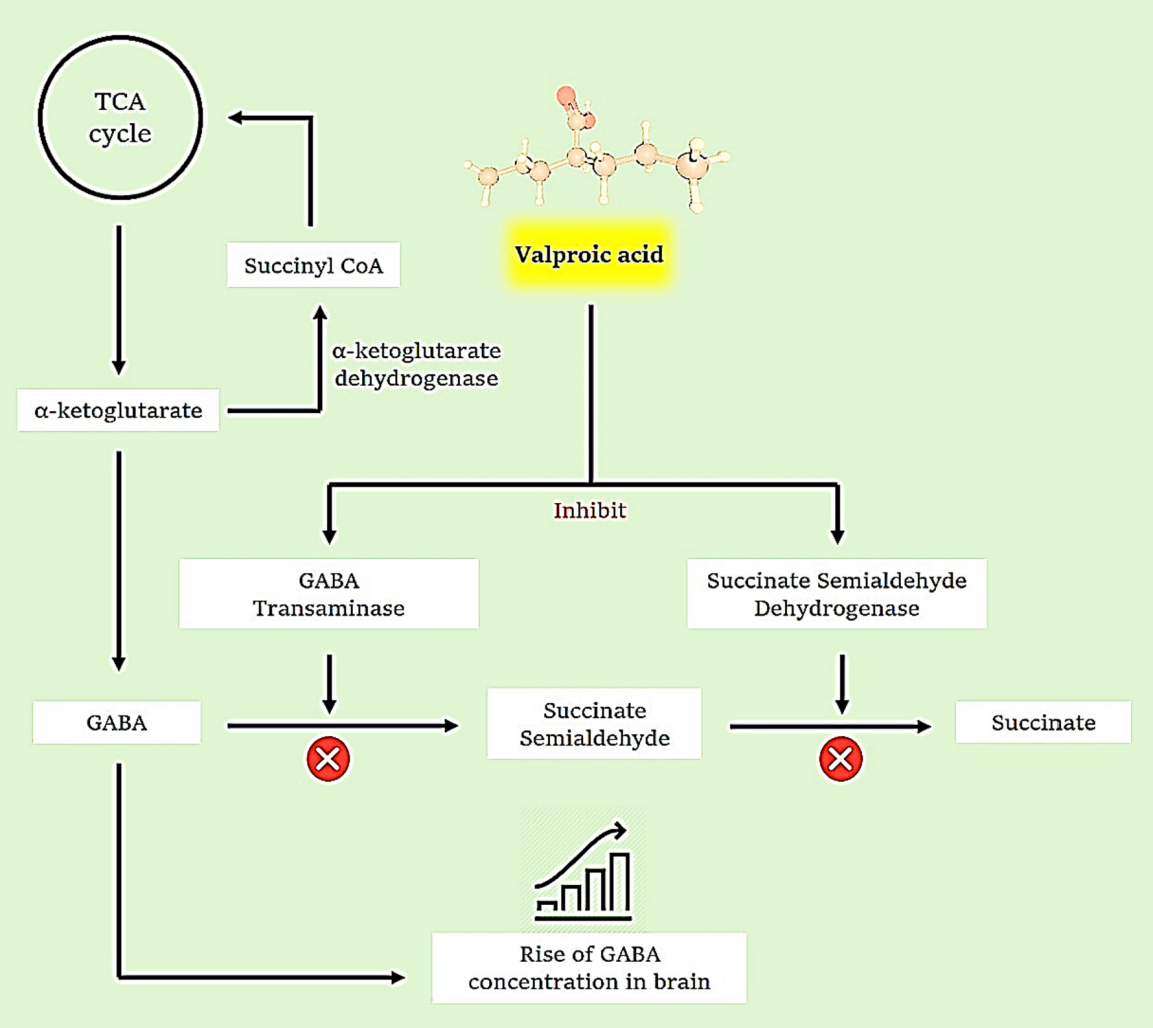
Valproic acid's mechanism of action. The metabolism of the GABA synthesis pathway by alfa-ketoglutarate dehydrogenase, GABA transaminase, and succinate dehydrogenase is depicted in the figure. Valproic acid suppresses two downstream catabolic enzymes in GABA metabolism, increasing GABA levels in the CNS.

Valproic acid exerts its pharmacological action in several ways, including increasing levels of GABA (γ aminobutyric acid) in the CNS, suppressing histone deacetylase, and blocking voltage-gated ion channels. Altered GABAergic inhibition is well-known pathophysiology of seizure start and progression, and regulating this route is a possible focus for anti-seizure medicines. GABA is produced by using α-ketoglutarate through the TCA cycle, then metabolized into succinate semialdehyde before being converted to succinate using GABA transaminase and succinate semialdehyde dehydrogenase. Previous research has demonstrated that valproic acid blocks GABA transaminase and succinate semialdehyde dehydrogenase, hence raising GABA levels by decreasing its breakdown (Fig. 4) (Rahman and Nguyen, 2021).

Valproic acid may also have antiepileptic effects by inhibiting high-frequency neuronal activity via voltage-gated calcium, sodium, and potassium channels. Valproic acid impacts nociception and the physiological phenomenon of aura by modifying GABA and/or neurotransmission mediated by glutamate. Valproic acid has been shown to decrease neurogenic inflammation in neuropathic pain via GABA-A receptor inhibition. HDAC has recently been proven to be blocked by valproic acid, specifically HDAC1, and also other HDAC. Histone deacetylase inhibitors may increase the interpretation of apoptosis and anticancer genes (Ghodke-Puranik et al., 2013). Valproic acid also impacts signaling systems such as the pathways of ERK and Wnt/Beta-Catenin, which interfere with arachidonate and inositol metabolism. The use of valproate influences the expression of numerous genes associated with cell survival, ion homeostasis, transcription control, signal transmission, and cytoskeletal changes. The underlying action of VPA in the treatment of all three indications described above can be explained by both immediate biochemical effects and long-term genetic implications (Löscher, 2002).

Following acute therapy with valproic acid, during chronic constant-rate delivery of the medication through osmotic minipumps, concentrations of VPA and its metabolites were assessed in cerebrospinal fluid, plasma and specific brain regions in rats and dogs. For the analysis of drugs and metabolites, very efficient gas chromatograph-mass spectrometer-computer techniques were employed. Of multiple VPA metabolites detected within the plasma of rats and dogs, only 2-en-VPA (2-propyl-2-pentenoic acid) was discovered in both species' brains. While 2-en-VPA buildup was observed in specific brain regions after extended therapy, the distribution of 2-en-VPA and VPA within the brain seemed generally uniform. When calculations are made on basis of whole-brain concentrations, 2-en-VPA's potency testing in mice revealed that the substance is roughly 1.3 times more effective as compared to parent medication. Even though 2-en-VPA showed plasma protein binding substantially higher than that of valproic acid, administration of this chemical to a dog revealed that its pharmacokinetics are identical to those of valproic acid. The findings imply that 2-en-VPA may considerably contribute to the anticonvulsant effects of (chronic) VPA therapy (Löscher and Nau, 1983).

## 4 Pharmacological applications

### 4.1 Valproic acid action on the cardiovascular system

**Fig. 5:**
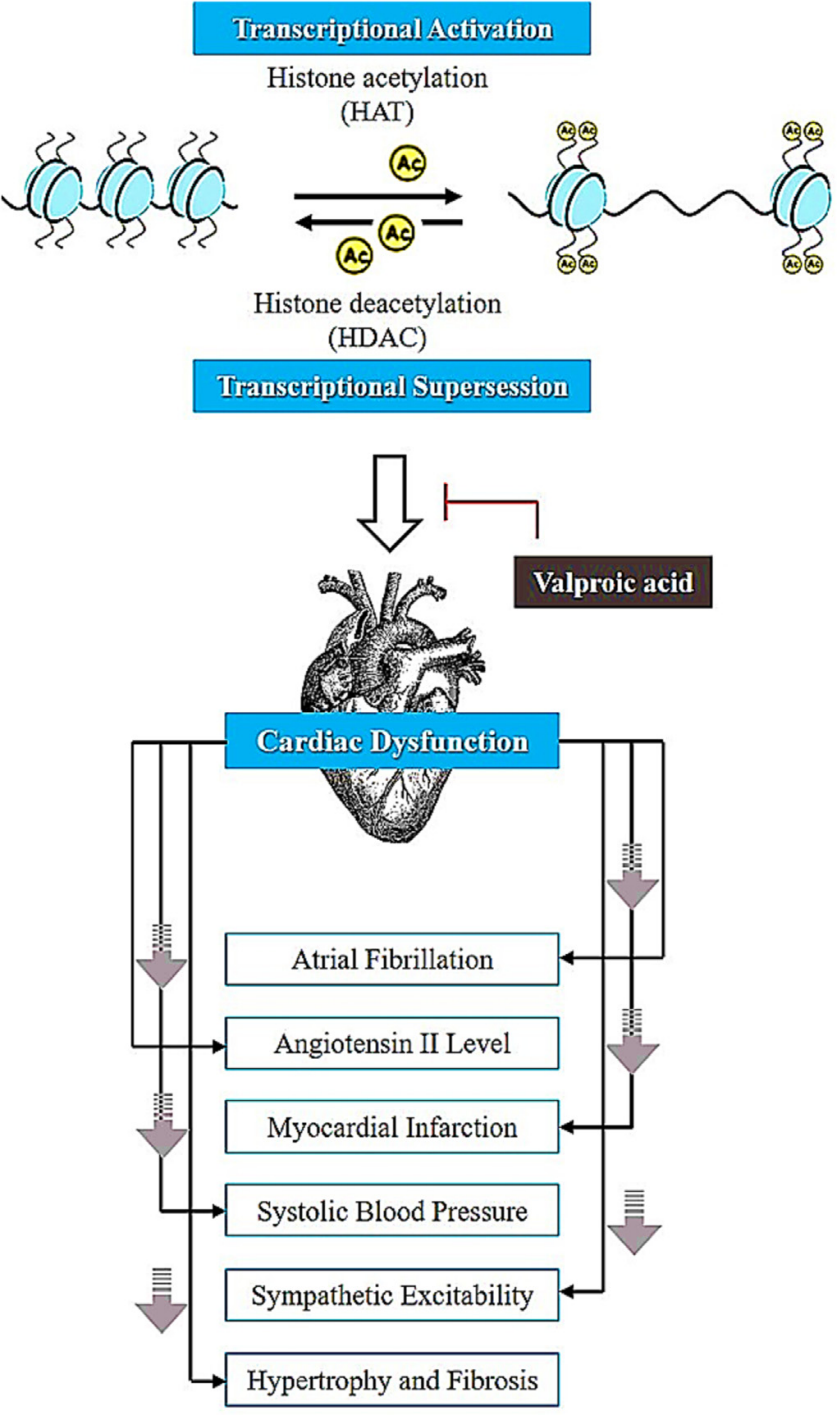
Valproic acid (VPA) has therapeutic potential in heart dysfunction. VPA works by inhibiting HDACs (histone deacetylases) activity, resulting in histone acetylation and chromatin structural relaxation, resulting in gene expression, and found to be completely effective in the treatment of enlarged heart, cardiac arrhythmia, cardiac hypertension, myocardial infarction, and fibrosis.

VPA demonstrated cardioprotective activity (Fig. 5) via its activity on histone deacetylases and is capable of influencing genomic administration. Li and his colleagues looked at how a rat model of renovascular hypertension helped to protect the heart. 400 mg/kg of valproic acid improved congestive heart failure, cardiac hypertrophy, and fibrosis by inhibiting the manifestation of HDAC8, HDAC2, TGF-β1 (transforming growth factor-β1), and CTGF (connective tissue growth factor) (Li et al., 2017). In spontaneously hypertensive rats, VPA was demonstrated to reduce myocardial fibrosis and hypertrophy by reducing histone deacetylases’ ability to give mineralocorticoid receptors an acetyl group (Kang et al., 2015). During the pressure overload experimental research, Yang et al. found that VPA at a dosage of 300 mg/kg once a day for 4 weeks, changed the way the left ventricle worked, made the sympathetic nervous system less active, and slowed down the heart's remodeling (Liu et al., 2017). 8 weeks of VPA treatment in transgenic mice reduced atrial fibrillation and remodeling. This had to do with a significant decrease in (i) fibrosis and dilation of atria, (ii) cardiomyocyte enlargement (iii) structural disarrangement in myocytes via modulating RhoA signaling or oxidative phosphorylation (Scholz et al., 2019). Tian et al. discovered that giving rats 250 mg/kg of valproic acid, decreased the size of myocardial infarcts by 50 % and raised systolic blood pressure by blocking histone deacetylase and increasing the expression of the Foxm1 gene, which led to more cardiomyocyte cells living (Tian et al., 2019). Another study found that after six days of treatment of valproic acid with a dosage of 300 mg/kg, there was a reduction in angiotensin II and a reduction in HDAC1 expression in a high-fat-diet rat model (Choi et al., 2017). Furthermore, VPA inhibited sepsis-induced cardiac dysfunction by increasing myocardial autophagy via enhancing the expression of PTEN and decreasing the AKT/mTOR pathway (Shi et al., 2019).

### 4.2 Neuroprotective role of VPA

**Fig. 6:**
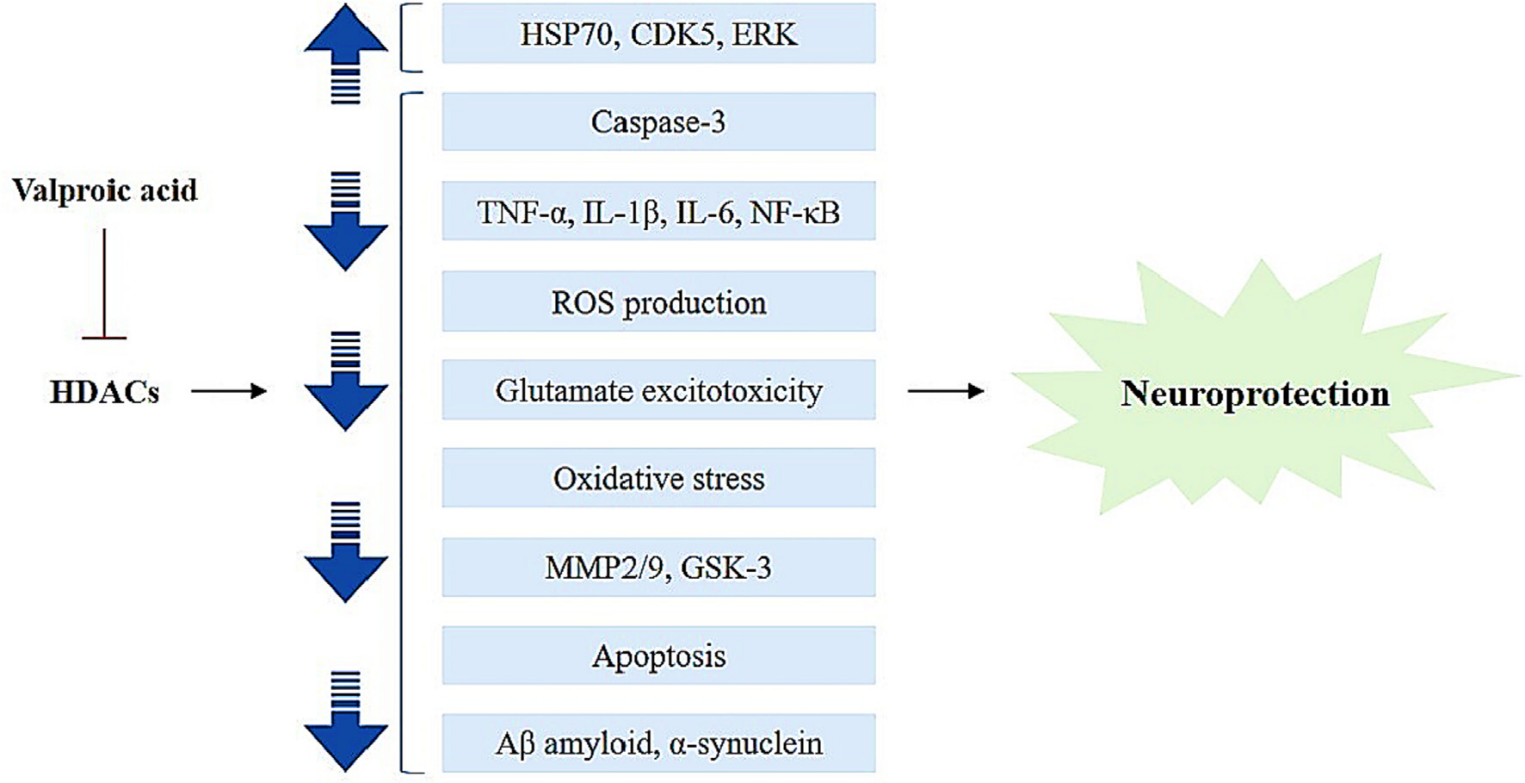
Valproic acid shows therapeutic potential for neurodegenerative illness. It blocks HDACs (histone deacetylases), resulting in anti-apoptotic transcriptional activation and activation of neural survival paths such as NF-κB, AKT, HSP70, ERK, CDK5, and restriction of caspase-3, IL-1β, IL-6, ROS, TNF-α, and various factors that lead to the death of neurons. HSP70: Heat Shock Protein 70; CDK5: Cyclin-Dependent Kinase 5; AKT: Enzyme serine/threonine-protein kinase; ERK: Extracellular Signal-regulated Kinases; MMP: Matrix metalloproteinases; IL-1β: Interleukin-1β; NF-κB: Nuclear Factor-κB; GSK: Glycogen Synthase Kinase; ROS: Reactive Oxygen Species; TNF-α: Tumor Necrosis Factor-α.

Neural rhythmic networks are involved in the processing of information, storage, and retrieval, which is important for memory and learning, executive function, and sensory perception. Thus, brain arrhythmias may have a disastrous influence on the operation of the circuit, which is believed to be the most important indication for various neurological illnesses. Furthermore, brain arrhythmias can serve as indicators for a wide range of brain diseases (Peña-Ortega, 2019). An imbalance in histone acetylation and, as a result, transcriptional dysfunction is linked to a wide range of neurodegenerative disorders (Fig. 6) (Sharma et al., 2019).

Valproic acid has been demonstrated to improve white matter remodeling, as well as neuron production following the stroke. VPA administration 28 days after the obstruction of the middle cerebral artery (on the investigational stroke model), significantly enhanced oligodendrocyte existence and oligodendrogenesis, showing higher levels of myelinated axon in an ischemic penumbra (Liu et al., 2012). Valproic acid showed damage to the brain inside a rat transitory focal cerebral ischemic model by blocking histone deacetylase and raising the HSP (heat shock protein) levels. Valproic acid therapy (300 mg/kg) immediately after ischemia, following the repeated injections every twelve hours, reduced the size of the infarct and the scores of neurological insufficiencies caused by ischemia. Caspase-3 stimulation was reduced as well (Ren et al., 2004). Angiogenesis and functional recovery following ischemic stroke in mice were enhanced by continuous treatment with valproic acid. They attributed these findings to VPA's capacity to decrease histone deacetylase, activation of HIF-1α (hypoxia-inducible factor-1α), as well as its related matrix metalloproteinase2/9 and proangiogenic VPF (vascular endothelial growth factor) (Wang et al., 2012). The advanced study demonstrated that VPA's neuroprotective impact in a rat brain ischemic model is due to its reduction of GSK-3 (glycogen synthase kinase-3) and HDAC (Silva et al., 2018). Furthermore, VPA demonstrated a neuroprotective impact on rats’ global transient ischemic model by lowering ROS generation and cerebral inflammation, as well as inhibition of HDAC and stimulation of HSP70 (Xuan et al., 2012).

In the 6-OHDA (6-hydroxydopamine) rat model of Parkinson's disease, Ximenes et al. discovered that VPA has neuroprotective properties. They found that rats who had 6-OHDA injected into their brains, increased IL1, IL6 and TNF release within the striatum, whereas inflammatory mediators were reduced by oral ingestion of valproic acid at a dose of 50 mg/kg and reduced degeneration of neurons within the striatum because of VPA's histone deacetylase inhibitory and anti-inflammatory effects (Ximenes et al., 2015). VPA also showed neuroprotective properties in Parkinson's disease induced by rotenone. Rotenone, a mitochondrial toxic chemical, damaged dopaminergic neurons, whereas continuing therapy with valproic acid lowered synuclein monoubiquitinated protein nuclear translocation dopamine-producing nerve cells, shielded neurons in the substantia nigra, and raised striatal dopamine and tyrosine hydroxylase levels. Valproic acid was capable of boosting the expression of neuroprotective genes by increasing the acetylation of histone H3 and suppressing the histone deacetylase enzyme (Monti et al., 2010). Using transgenic mice with LRRK2 R1441G, valproic acid reduced neuron damage and Parkinson's disease-like symptoms. Within substantia nigra of mice, VPA therapy amplified acetylation of histone and neurons count containing tyrosine hydroxylase. It reduced ionized calcium-binding adaptor molecule 1-stimulated microglia and pro-inflammatory mediator levels, alleviating both motor and non-motor impairments in Parkinson's disease (Kim et al., 2019). VPA rescued dopaminergic SH-SY5Y cells from MPP + -induced neurotoxicity in vitro. Dopaminergic cells were less likely to die when treated with VPA before being exposed to MPP +. This was done by reducing the production of reactive oxygen species by activating the ERK signaling path and the CDK5/p35 cascade (Muangsab et al., 2019). Leng et al. demonstrated that valproic acid might generate α-synuclein cerebral cortical neurons of rats by suppression of histone deacetylase, which attributed to its neuroprotective role from excitotoxicity of glutamate (Leng and Chuang, 2006). In a Huntington's disease model, VPA enhanced survival and aberrant motor activity. Daily injection of VPA enhanced the longevity of genetically modified mice and recovered their inability to perform involuntary locomotion while having no discernible negative impact on their performance. Administration of valproic acid improved TBI-induced autophagic flux blockade and enhanced stimulation of autophagy, preventing brain cell apoptosis and influencing the activation of microglia and phenotypic divergence to boost post-traumatic restoration and act as an efficient treatment for brain injury after a trauma (Zheng et al., 2020).

Similarly, one other study found that valproic acid has neuroprotective and anti-apoptotic impacts against traumatic brain injury via increasing the expression of AKT and ERK, and that these signaling pathways facilitate VPA neuroprotection (Zhang et al., 2014). VPA displayed anti-apoptotic and neuroprotective properties in primary cortical neurons and SH-SY5Y cells via decreasing stress in the endoplasmic reticulum and boosting the activity of AKT. VPA protects rats' brains from ischemia-reperfusion injury. VPA therapy changes the expression of Bcl2 and suppresses the activation of caspase-3, suggesting that VPA may protect neurons from apoptosis (Garay and Loureiro, 2015). Furthermore, in an experiment in which valproic acid was administered to pregnant rats, it was found to prevent apoptosis by increasing Bcl2 expression and decreasing the expression of developing neurons. This mechanism, in fact, explains the harmful consequences of VPA (teratogenicity) during fetal exposure (Go et al., 2011).

### 4.3 Anticancer role of VPA

DNA methylation and Histone deacetylation are mechanisms implicated in chemotherapy resistance by protecting malignant cells by silencing critical anti-tumor genes. HDAC inhibitors are thought to work by stimulating or de-repressing suppressed anti-oncogenes. Histone deacetylases have a significant effect on how chromatin changes and how genes work, enabling the use of these inhibitors to learning more about cancer (Bai et al., 2019). VPA increased the antiproliferative action of melatonin in MCF-7 cells of breast cancer by increasing melatonin receptor MT1 expression (Jawed et al., 2007). Terranova et al. discovered that VPA at low concentrations increased protein expression and thymidine phosphorylase transcript in breast cancer cells with regard to time and frequency of use. The same impact, however, has not been observed with non-cancerous breast cell line MCF-10. Thus, discovered that valproic acid increased the activity of capecitabine in breast cancer cells after suppressing histone deacetylase 3; although, this impact was eliminated in cells lacking thymidine phosphorylase (Terranova-Barberio et al., 2016).

**Fig. 7:**
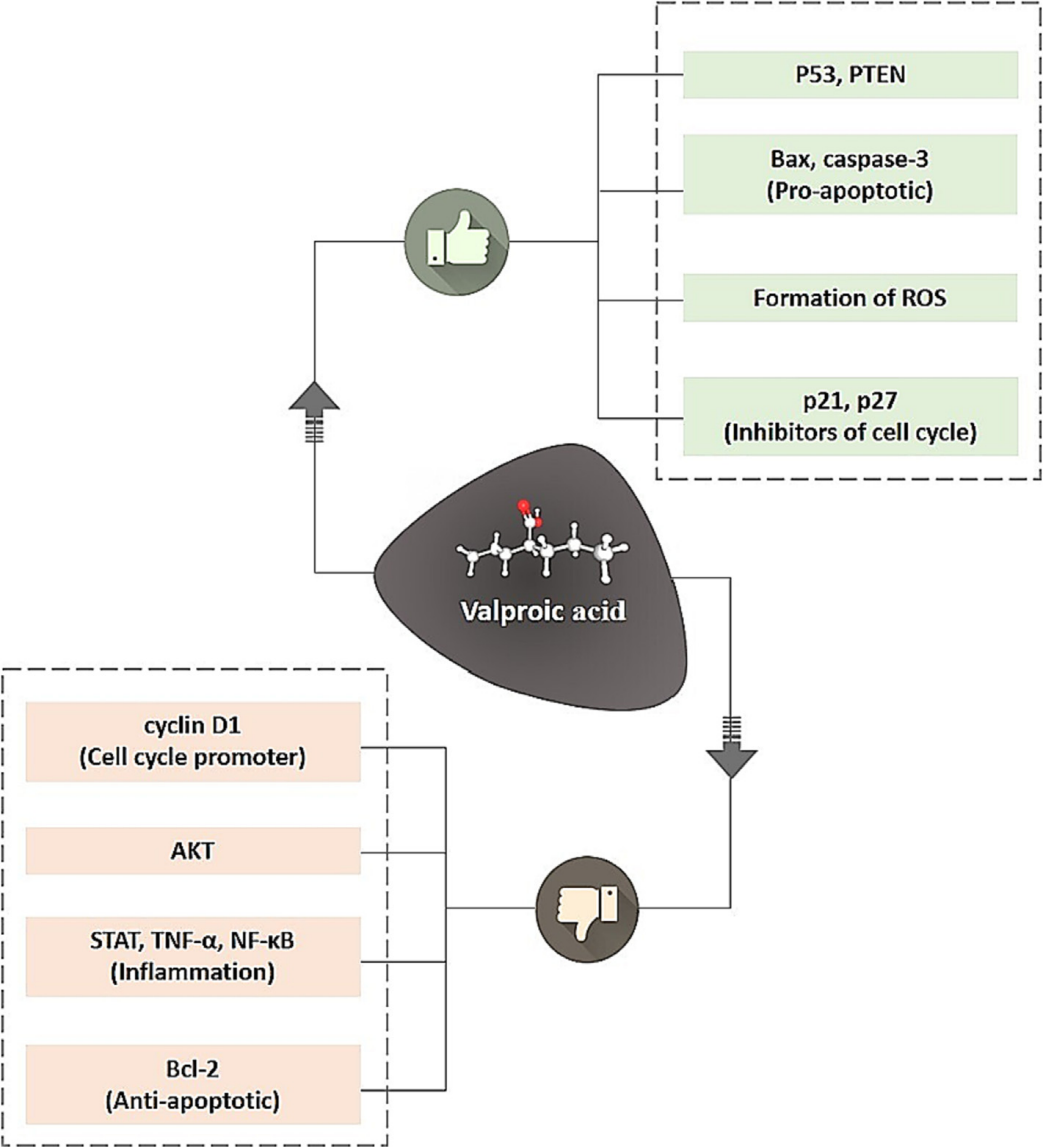
The beneficial effects of VPA on cancer. VPA influences the prevalence of cancer after activation of Bax, PTEN, p53, ROS, p21, p27, and caspase-3, resulting in apoptosis, growth, and cycle halt. Valproic acid suppresses STAT, AKT, cyclin D1, Bcl2, NF- κB, and TNF- α, all of these contribute to stopping apoptosis, so cancer cells can live longer. PTEN: Phosphatase and Tensin homolog deleted on chromosome 10; Bax: Bcl2 associated X; ROS: Reactive Oxygen Species; caspase-3: cysteine aspartic protease-3; TNF-α: Tumor Necrosis Factor-α; STAT: Signal Transducers and Activators Transcription; NF-κB: Nuclear Factor-κB; AKT: Enzyme serine/threonine-protein kinase; Bcl2: B cell lymphoma 2.

In vitro and in vivo tests showed that valproic acid and arsenic trioxide worked together to kill NCI-H460 and NCI-H1299 cancer cells in the lungs. This combination effectively inhibited the lung cancer cells proliferation by stopping the G2 or M phase of the cell cycle and triggering caspase-dependent death (Park et al., 2020). The effect of valproic acid and gemcitabine together on pancreatic cancer cells was also investigated. Valproic acid was found to boost the susceptibility of gemcitabine to pancreatic tumor cells in a dose-dependent manner. Surprisingly, a small amount of valproic acid enhanced the intrusion of pancreatic carcinoma cells which had already been shown to make gemcitabine motility. Also, a small dose of valproic acid turned on the ROS/AKT/STAT3/Bmi1 pathway, which made it easier for pancreatic tumors caused by gemcitabine to move and grow into healthy tissue (Li et al., 2019). Similarly, a study examined how VPA combined with P276-00 (a cyclin-dependent kinase inhibitor) affected the cancer of lung cells. This study found that either evaluated at p53 + lung cell cancer or p53- lung cell cancer, this combination is synergistic, with a substantial reduction during colony formation, tumor inhibitor proteins expression is augmented, including p21, p27, and p53, also decrease in surviving proteins such as cyclin D1 and Bcl2 expression (Shirsath et al., 2013). VPA was tested for anticancer efficacy on rat hepatoma FaO cell line (Fig. 7). Depending on the dose, it induced apoptosis in the hepatocytes of rats. Caspase-11 transcription and caspase-3 enzyme activity were both increased, suggesting that caspase may play a role in FaO cell death via valproic acid-mediated apoptosis (Phillips et al., 2003). Metformin and VPA together increased apoptosis and inhibited LNCaP and prostate PC-3 cancer cells by activating the p53 signaling pathway in prostate cancer (Lin et al., 2019). In metformin-resistant kidney cancer cells, VPA was demonstrated to have a synergistic impact with metformin. This impact was linked to VPA's capacity to improve H3 acetylation (Wei et al., 2018). The anticancer activity of VPA on tumor progression and Notch1 overexpression was investigated in a thyroid cancer cell line. VPA was found to be able to promote expression of Notch1, apoptosis is accelerated, and inhibited carcinoma cell proliferation (Guenter et al., 2021).

Michaelis et al. examined the impact of VPA and IFN-α combination on neuroblastoma cell lines including UKF-NB-3 and NB-2. They discovered that the combination of VPA and IFN-α reduced the development of UKF-NB-3 xenograft tumors synergistically in nude mice and enhanced complete recovery in two animals out of a total of six, whereas single treatment only inhibited tumor growth (Michaelis et al., 2004). When VPA was given to gastric tumor cells, it stopped HDAC1/2 from working and made autophagy better. This made apoptosis happen more quickly by blocking HDAC1/PTEN/AKT signaling pathway (Sun et al., 2020). The combined therapy of VPA and oridonin which is a diterpenoid, suppressed the metastasis and proliferation of HL-60 leukemic cells, according to Li and Ma's research. HL-60 leukemic cells were treated with oridonin, which stimulated the intrinsic pathways of apoptosis and decreased the Bcl2/Bax ratio (Li and Ma, 2019). VPA also improved the GANT61impact in inhibiting the growth of numerous myeloma cells in a time and dose-dependent manner by inhibiting the upregulation of the HES-1 GLI1 and PTCH1 signaling pathway (Zhang et al., 2020). There were clinical trials of VPA's anticancer properties as well as experiments, which is unusual. As an example, in a phase I/II clinical study, VPA in combination with karenitecin, a topoisomerase I inhibitor, 47 percent of melanoma patients with stable disease had average progression-free longevity of 10.3 weeks, while 34 percent of melanoma patients on single treatment had stable disease with an average progression-free longevity of 7.9 weeks (Daud et al., 2009). In a clinical trial involving patients with advanced melanoma, VPA increased cancer cells' sensitivity to chemotherapy, caused growth arrest, induced apoptosis in glioma cells, and enhanced survival rates (Rocca et al., 2009).

Patients (11 of 36) having acute myeloid leukemia (with an average age of 77) who were not suitable for standard intensive chemotherapy responded to valproic acid, a reduced dosage of cytarabine, and all-trans retinoic acid in a clinical trial conducted on them. A low incidence of side effects and two patients going into full remission was found to be associated with the drug combination (Fredly et al., 2013). VPA may be able to decrease the highest tolerated temsirolimus dosage in pediatric patients having solid tumors, according to a study published in 2013. Temsirolimus's blocking effect on regular CD34 + hematopoietic precursors was maintained with only minimal toxicities in the combined treatment, proving their significance and safety in the treatment of B lymphoma (Coulter et al., 2013). In a phase I study, children with solid tumors that didn't respond to VPA at concentrations of 75–100 g/ml were able to handle it well. Half of the people who were studied had histones that were too acetylated in their peripheral blood mononuclear cells (Su et al., 2011). A phase II clinical trial was conducted to evaluate the benefits of incorporating VPA to each week paclitaxel in gastric cancer patients. In this survey, there was no significant difference between each week of paclitaxel (median on the whole survival of 9.8 months) and each week of paclitaxel in combination with valproic acid (median on the whole survival of 8.7 months) (Fushida et al., 2016). The intensity of myeloid-derived suppressor cells was decreased as a result of treatment with VPA, which was accompanied by a reduction in the expression of TLR4 mRNA (Xie et al., 2018).

### 4.4 Antimicrobial effect of VPA

Resistance to antibiotics is a major public health issue, particularly in underdeveloped nations where infectious illnesses continue to be a major reason for human death. Antibiotics are either cytostatic or cytotoxic to harmful germs, assisting the natural defense system of the body in their eradication (Levy and Marshall, 2004). VPA has a strong antifungal effect on resistant and sensitive Candida albicans in a pH-dependent manner. VPA had a strong anti-biofilm impact, and reduced damage to the vaginal epithelial cells because of Candida albicans, enhancing the action of antifungal drugs like terbinafine. VPA's ability to kill fungi was associated with its capability to disrupt the integrity of fungal vacuoles (Chaillot et al., 2017). The bactericidal activity of numerous anticonvulsant medications, including VPA, was investigated by Nathiya et al. At 100 g/ml, VPA was highly efficient against Proteus vulgaris and Staphylococcus aureus, but other examined species needed more than 200 g/ml. These isolated bacteria were resistant to gabapentin and carbamazepine. As a result, it was found that valproic acid, amongst other anticonvulsants, showed a distinct antibacterial activity and could help treat specific bacterial infections (Singh et al., 2021).

### 4.5 Interaction of VPA with different drugs

Acetaminophen, ertapenem, propoxyphene, cilastatin/imipenem, aspirin/caffeine, doripenem, meropenem, lamotrigine, sodium oxybate, sodium benzoate, vorinostat, and sodium phenylacetate, are the most common medication interactions. Caffeine, aspirin, and other salicylates can block VPA clearance, bringing its count of serum-free cells up to fourfold (Verrotti et al., 2019), this may assist to decrease the regular intake of VPA, but when combined with aspirin, it also increases its cytotoxicity. Antibiotics such as imipenem, doripenem, meropenem, panipenem, and ertapenem reduce blood valproic acid concentrations most likely through boosting VPA metabolism to VPA glucuronide and also the kidneys' ability to get rid of valproic acid glucuronide and the gut's ability to absorb VPA (Koeck et al., 2021). VPA has also been found to remarkably raise lamotrigine levels in plasma (to double its half-life), presumably causing the severe and a potentially fatal rash, such as toxic epidermal necrolysis and Stevens-Johnson syndrome, as well as disabling ataxia and tremors, which are known as lamotrigine's secondary effects (Arif et al., 2007). Propoxyphene-VPA interaction is linked to 60 % more hip fractures in the elderly, possibly because of additive psychomotor impairment (Jagtap, 2020). Furthermore, propoxyphene, according to various medical websites, can cause additional respiratory or CNS depressing effects, as well as deaths caused by drugs, especially when combined with VPA in people with mental illness or drug addiction. Because of urea cycle enzyme shortages, VPA reduces the healing efficacy of sodium phenylacetate and sodium benzoate in the cure of acute form of hyperammonemia (Nicolai et al., 2007). Other CNS depressants, such as the VPA, can potentially increase sodium oxybate's (gamma-Hydroxybutyrate, GHB) therapeutic effect on the respiratory system and CNS, however, the production of medium and short-chain monocarboxylic acids is greatly diminished, GHB brain influx ranges between 35 % and 90 %. (Trombley et al., 2019) consequently, its effectiveness is diminished. Valproic acid (also other HDACi) coadministration with vorinostat SAHA (suberoylanilide hydroxamic acid) can worsen gastrointestinal bleeding and thrombocytopenia carried on by SAHA treatment (Spartalis et al., 2019).

### 4.6 Valproic acid side effects

The most prevalent general adversative impacts of VPA therapy are vomiting, heartburn, and nausea, which are less common when enteric-coated versions are used (Schäfer and Brandl, 2020). Some general and most prevalent side effects observed during VPA treatment are mentioned in Table 1. 10 % of the population individuals report dermatological side effects (alopecia/rash), dosage-related tremors, and neurological side effects including ataxia, sleepiness, and irritability. Besides these side effects, valproic acid is a teratogen in humans, which means it can cause birth defects linked to an increased incidence of spina bifida aperta, a prenatal disease characterized by abnormal closure of posterior neural tube, and also heart deformities, cleft palate, and limb anomalies. In rodents, Valproic acid is also a teratogen. VPA also has an impact on reproductive and endocrine function. There is some disagreement over whether VPA causes symptoms linked with the polycystic ovarian syndrome (PCOS) in women (Forrest et al., 2020). Besides affecting the reproductive system, VPA has been shown to directly impact steroidogenesis in cultured cells (Nelson-DeGrave et al., 2004). VPA's effects are not restricted to women; males who have been exposed to VPA have experienced a decrease in reproductive function as well as a decrease in testicular volume (Eklioglu and Ilgin, 2022). Rodent studies also found a decrease in epididymal weight, but no changes in fertility (Maneenin et al., 2018).

**Table 1:** A list of categorized adverse effects observed with Valproic acid medication.

**Adverse effects**	**Pervasiveness**	**Reference**
Gain of weight	14 % weight class change	(Grosso et al., 2009)
Parkinsonism	1.37–5.04 %	(Jamora et al., 2007)
Aplastic anemia	9-fold rise	(Handoko et al., 2006)
von Wilbrant disease	67 % prevalence in children and 16 % in adults	(Gerstner et al., 2006)
Deficiency of factor XIII	4 %	(Gerstner et al., 2006)
Decrease in fertility rate	25 %	(Ornoy et al., 2020)
The decline in duration of sleep time after treatment conclusion	15 to 45 min/day	(Schmitt et al., 2009)
Hepatotoxicity in children	0.16 %	(Hani and Husain, 2018)

Valproic acid and other psychotropic medications are also known to cause weight gain, which has been the topic of numerous recent studies. In a three-year relative trial comparing valproic acid with CBZ to cure epilepsy, gaining weight has been the most frequent side effect associated with valproic acid medication in adults and children (Moavero et al., 2018). The specific VPA-induced mechanism for weight gain is unidentified. Medical condition, gender, VPA dose, serum concentration, and age, along with the family background of weight problems, are substantially not connected to weight gain related to VPA treatment. Weight gain is the well-known adverse impact of valproic acid treatment to cure diseases of CNS, leading to non-adherence and elevated damage to health issues related to weight gain, but trials investigating the efficiency of Valproic acid in cancer patients may also uncover a possible unrevealed advantage (Tsai et al., 2018).

Patients using VPA may experience metabolic abnormalities such as hyperammonemia and hypocarnitinemia. These problems primarily affect patients who already have metabolic abnormalities that are probably being hidden by the increased load of valproic acid metabolism. The most common side effects of VPA are thrombocytopenia and suppression of platelet aggregation, which happen in 12 percent of patients; nonetheless, most cases of thrombocytopenia are mild, showing platelet counts ranging from∼100–150 × 103/mm3 (Conley et al., 2001). VPA-induced hemorrhagic pancreatitis and reversible idiopathic hepatitis are unique and idiosyncratic non-dosage-related adverse impacts. These incidents are tremendously uncommon but typically fatal; such as, 29 people died in the United States from 1987 to 1993 as a result of hepatotoxicity from VPA. Valproic acid derivatives are being researched to find agents which are more efficient, less hepatotoxic, and least teratogenic (Mishra et al., 2021).

## 5 VPA-induced toxicity

Teratogenicity, reproductive toxicity, nephrotoxicity, and hepatotoxicity are some of the harmful consequences of VPA, which have now been thoroughly examined.

### 5.1 Teratogenicity

VPA's teratogenic effects are thought to begin in the earliest stages of organogenesis when the closure of the neural tube is extremely sensitive to its metabolite (Brotzmann et al., 2021). Cardiac aberrations, neural tube defects (NTDs) including spina bifida, cleft lip or palate, skeletal or limb distortion, behavioral issues, lower cognitive function, and diminished verbal intelligence with autism spectrum disorder (ASD) communication problems are among the major anomalies identified. Numerous hypotheses indicate that drug buildup in the fetus, increased oxidative stress in the fetus particularly within the brain, inhibition of HDAC (histone deacetylase), and folate antagonism, are among the molecular procedures of VPA's teratogenic action. HDAC suppression and the formation of ROS (reactive oxygen species) are particularly important during the first three months of pregnancy when DNA dysregulation has the greatest impact on fetal organogenesis (Lloyd, 2013). VPA deposits in the embryo's circulation in the majority of the cases, reaching higher concentrations than those present in the blood of the mother; which may be accountable for the toxic effects and a higher threat (about threefold) of teratogenicity (Vajda, 2012).

On the other hand, because the fetus' defense system is immature in the first three months of pregnancy, ROS produced by bioactivation of VPA impede ETC (electron transport chain) for energy creation, limiting the detoxification process and boosting interactions of ROS with biomolecules such as DNA (Lloyd, 2013). The distinctive expression of multiple genes engaged in DSB (DNA double-strand break) repair (Brca2, Rad51, and Brca1) after exposure to valproic acid in post-implantation mouse embryos has been demonstrated to promote homologous recombination and to generate DSBs (Lamparter and Winn, 2014). Enhanced expression by cleaved caspase 3, caspase 9, poly (ADP-ribose) polymerase (PARP), and p53 target genes may contribute to increased apoptosis (Paradis and Hales, 2015). Also, the VPA has been shown to aggravate folate deficit by blocking critical enzymes in the metabolic pathway of folate during embryogenesis, causing teratogenicity. Gene expression and its regulation by epigenetics, oxidative stress induction, hyperhomocysteinemia, and interruption of essential proteins are all associated with negative effects. Folate insufficiency has been linked to poor homocysteine metabolism, which results in NF-κB (nuclear factor-kappa B) activation and induction of apoptosis through the action of oxidative stress (Lloyd, 2013). The hydrolytic excision of DNA acetyl groups is restricted by Valproic acid (or its bioactive components) binding directly to the active site of HDACs. Multiple transcription factors, including Hoxa1 and Bcl-2 (B-cell lymphoma protein 2), can affect the transcription of possibly hazardous genes in the open and uncondensed chromatin, which increase apoptosis regulation while decreasing morphogenesis (essential for differentiation of organ) and cellular proliferation, respectively (Ornoy, 2009).

### 5.2 Hepatotoxicity

The suggested mechanism of hepatotoxicity induced by VPA supports the formation of reactive intermediates to impede β-oxidation, resulting in steatosis, and hence causes mitochondrial dysfunction. VPA-induced mitochondrial dysfunction is associated with lower consumption of oxygen and levels of ATP (adenosine triphosphate), as well as lower DNA polymerase gamma expression, which is participating in mtDNA (mitochondrial DNA) replication and repair (Li et al., 2015), reduced potential of mitochondrial membrane of the liver, respiratory control ratios, and enhanced swelling of mitochondria because of calcium flux alterations (Komulainen et al., 2015). VPA causes over-regulation of the cluster of differentiation 36 (CD36), an essential fatty acid transporter, and diacylglycerol acyltransferase 2 (DGAT2) in liver cells when it causes steatosis or the buildup of fat inside cells. This made the MEK/ERK signaling pathway less effective, which led to more DGAT2 expression and more triglyceride synthesis (Bai et al., 2017). More research into how VPA causes steatosis shows that patterns of methylation change in mitochondrial DNA (mtDNA) and nuclear DNA (nDNA), which may play a big role in the malfunctioning of mitochondria and the progression of steatosis. Mitochondrial dysfunction induces rapid production of reactive oxygen species (ROS), which is most probably triggered by VPA-directed inception of the mitochondrial permeability transition (MPT) pore, discharge of cytochrome *c* from mitochondria, and caspase 9 activation, all of which result in mitochondria-directed apoptosis (Zhang et al., 2016).

### 5.3 Reprotoxicity

In humans, VPA is said to cause endocrine abnormalities in both genders. VPA has been shown to impact steroidogenesis within ovarian follicular cells in female rats, preventing testosterone conversion to estradiol (Sveberg Røste et al., 2002). Other investigations have found that a decrease in estradiol levels leads to a considerable increase in the testosterone/estradiol ratio. Females' levels of follicle-stimulating hormone (FSH), luteinizing hormone (LH), and progesterone have also been found to be lower (Ibrahim et al., 2019). Decreased androgen receptor levels in the testis epididymis and testis have been linked to increased FSH and LH levels in males (Iamsaard et al., 2017). Hyperandrogenism, menstrual problems, polycystic ovarian syndrome or polycystic ovary, and ovarian failure are all linked to VPA use in women. VPA therapy causes anomalies in sperm motility, androgen levels of blood, and erectile dysfunction in men (Verrotti et al., 2016). The drug's effect is predominantly felt at the gonadal level, based on these studies of changes in reproductive hormone levels. VPA-induced reproductive toxicity has also been linked to oxidative stress, which results in apoptosis both in medium and small testes and follicles, along with the damage to sperm DNA (Khan et al., 2011). It has also been shown that redox responsive transcription factor NF-κB, which mediates inflammation, is involved (Savran et al., 2020).

### 5.4 Nephrotoxicity

VPA causes oxidative stress, mitochondrial deficiencies, carnitine shortage, fibrosis, and inflammation in mice renal tissue, according to experimental and clinical investigations (Hamed, 2017). Elevated MDA (malondialdehyde) levels, a lipid peroxidation marker; activity of xanthine oxidase and protein carbonyl, oxidative stress markers; decline in glutathione concentration and decrease in levels of thiol (non-protein), antioxidant markers; changed actions of GST (glutathione-S-transferase), GR (glutathione reductase), and GPX, enzymes for glutathione metabolism (Gezginci-Oktayoglu et al., 2016). Mitochondrial deficiencies, including impaired succinate dehydrogenase activity (SDA) of mitochondria, ATP, glutathione, mitochondrial permeability transition (MPP) pore, and elevated ROS of mitochondria and peroxidation of lipids, are additional postulated causes of VPA-induced kidney injury (Heidari et al., 2018).

Moreover, an increase in IL-1β, TNF-α, IFN-γ, expressions of inducible nitric oxide synthase (iNOS), levels of monocyte chemoattractant protein-1, NF-κB/p65, and activity of adenosine deaminase, showing inflammation, and altering arginase activity, and collagen-1 levels in renal tissues. VPA also boosted caspase-3 expression and triggered the notch signaling cascade. (Gad, 2018). In other investigations, VPA caused significant chromosomal abnormalities, mitotic index changes, and histological changes in kidney tissue, indicating its genotoxic potential (Galaly et al., 2014). Fanconi syndrome has been documented in association with valproic acid therapy, particularly in epileptic children, and is caused by VPA-induced malfunction of proximal renal tubules (Adewole et al., 2021).

## 6 Conclusion

VPA is a chemical that has many different effects on different tissues, diseases, and patient profiles. Almost 20 % of the transcriptome can be altered by this chemical in tissue-specific ways. As a result, it is currently exceedingly difficult to predict all of the side impacts as a result of mono- or polytherapy treatments. On the one side, valproic acid has been utilized for several years to cure epilepsy-related convulsive seizures, as a mood stabilizer, and to treat schizophrenia. VPA has lately been tested in clinical studies for diverse disorders of the nervous system and diseases, including migraine, and addiction. Its effectiveness in treating brain abnormalities is the most well-known. Controlling expression of oncogenes or anti-oncogenes, on the other side, could be a lucrative tactic in the treatment of numerous types of cancer. VPA appears to have certain unquestionable properties in this regard, including cell death elevation (mostly apoptotic) and differentiation, as well as suppression of proliferation. Surprisingly, healthy cells appear to be less responsive to these features. Given all of these findings, as well as the growing number of clinical trials employing VPA, it's quite probable that this compound will be used in a variety of medicines in the coming days. Nonetheless, most papers on the therapeutic efficacy of VPA also indicate the possibility of treatment-related adverse effects. As a result, various adverse effects have been recorded, such as coagulopathies, aplastic anemia, teratogenic consequences, and hepatotoxicity. The application of VPA in a therapeutic context is contraindicated in several cases, including hyperhomocysteinemia or coagulopathy, as well as pregnancy. The balance between therapeutic possibilities and significant side effects found after the application of valproic acid to encourage the positive benefits will turn out to be a difficult and complex clinical management challenge.

## Declaration of Competing Interest

The authors declare that they have no known competing financial interests or personal relationships that could have appeared to influence the work reported in this article.

## References

[b0005] Adewole K.E., Attah A.F., Osawe S.O. (2021). Exploring phytotherapeutic approach in the management of valproic acid-induced toxicity. Adv. Tradit. Med..

[b0010] Argikar U.A., Remmel R.P. (2009). Effect of aging on glucuronidation of valproic acid in human liver microsomes and the role of UDP-glucuronosyltransferase UGT1A4, UGT1A8, and UGT1A10. Drug Metab. Dispos..

[b0015] Arif H., Buchsbaum R., Weintraub D., Koyfman S., Salas-Humara C., Bazil C.W., Resor S.R., Hirsch L.J. (2007). Comparison and predictors of rash associated with 15 antiepileptic drugs. Neurology.

[b0020] Bai Y., Ahmad D., Wang T., Cui G., Li W. (2019). Research advances in the use of histone deacetylase inhibitors for epigenetic targeting of cancer. Curr. Top. Med. Chem..

[b0025] Bai X., Hong W., Cai P., Chen Y., Xu C., Cao D., Yu W., Zhao Z., Huang M., Jin J. (2017). Valproate induced hepatic steatosis by enhanced fatty acid uptake and triglyceride synthesis. Toxicol. Appl. Pharmacol..

[b0030] Baumgartner T.R., Elger C.E. (2020). Anti-convulsant agents: valproic acid. NeuroPsychopharmacother..

[b0035] Beckner R.R. (1979). Drug Evaluation Data: Valproic Acid (Depakene®-Abbott). Drug Intell. Clin. Pharm..

[b0040] Braconnier L., Simonnet A.J., Houard M., Benzidi Y., Robriquet L., Douillard C., Jourdain M. (2018). Encéphalopathie hyperammoniémique en Réanimation adulte: à propos de deux observations cliniques. Médecine Intensive Réanimation.

[b0045] Bradbury C.A., Khanim F.L., Hayden R., Bunce C.M., White D.A., Drayson M.T., Craddock C., Turner B.M. (2005). Histone deacetylases in acute myeloid leukaemia show a distinctive pattern of expression that changes selectively in response to deacetylase inhibitors. Leukemia.

[b0050] Brotzmann K., Wolterbeek A., Kroese D., Braunbeck T. (2021). Neurotoxic effects in zebrafish embryos by valproic acid and nine of its analogues: the fish-mouse connection?. Arch. Toxicol..

[b0055] Chaillot J., Tebbji F., García C., Wurtele H., Pelletier R., Sellam A. (2017). pH-dependant antifungal activity of valproic acid against the human fungal pathogen Candida albicans. Front. Microbiol..

[b0060] Chateauvieux S., Morceau F., Dicato M., Diederich M. (2010). Molecular and therapeutic potential and toxicity of valproic acid. J. Biomed. Biotechnol..

[b0065] Choi J., Park S., Kwon T.K., Sohn S.I., Park K.M., Kim J.I. (2017). Role of the histone deacetylase inhibitor valproic acid in high-fat diet-induced hypertension via inhibition of HDAC1/angiotensin II axis. Int. J. Obes..

[b0070] Chopra A., Kolla B.P., Mansukhani M.P., Netzel P., Frye M.A. (2012). Valproate-induced hyperammonemic encephalopathy: an update on risk factors, clinical correlates and management. Gen. Hosp. Psychiatry.

[b0075] Chou H.-F., Yang R.-C., Chen C.Y., Jong Y.-J. (2008). Valproate-induced hyperammonemic encephalopathy. Pediatr. Neonatol..

[b0080] Chung J., Cho J., Yu K., Kim J., Lim K.S., Sohn D., Shin S., Jang I. (2008). Pharmacokinetic and pharmacodynamic interaction of lorazepam and valproic acid in relation to UGT2B7 genetic polymorphism in healthy subjects. Clin. Pharmacol. Ther..

[b0085] Conley E.L., Coley K.C., Pollock B.G., DaPos S.V., Maxwell R., Branch R.A. (2001). Prevalence and risk of thrombocytopenia with valproic acid: experience at a psychiatric teaching hospital. Pharmacother. J. Hum. Pharmacol. Drug Ther..

[b0090] Coulter D.W., Walko C., Patel J., Moats-Staats B.M., McFadden A., Smith S.V., Khan W.A., Bridges A.S., Deal A.M., Oesterheld J. (2013). Valproic acid reduces the tolerability of temsirolimus in children with solid tumors. Anticancer. Drugs.

[b0095] Daud A.I., Dawson J., DeConti R.C., Bicaku E., Marchion D., Bastien S., Hausheer F.A., Lush R., Neuger A., Sullivan D.M. (2009). Potentiation of a topoisomerase I inhibitor, karenitecin, by the histone deacetylase inhibitor valproic acid in melanoma: translational and phase I/II clinical trial. Clin. Cancer Res..

[b0100] Eklioglu O.A., Ilgin S. (2022). Adverse effects of antiepileptic drugs on hormones of the hypothalamic-pituitary-gonadal axis in males: a review. Toxicology.

[b0105] Ethell B.T., Anderson G.D., Burchell B. (2003). The effect of valproic acid on drug and steroid glucuronidation by expressed human UDP-glucuronosyltransferases. Biochem. Pharmacol..

[b0110] Finsterer J., Zarrouk Mahjoub S. (2012). Epilepsy in mitochondrial disorders. Seizure.

[b0115] Forrest L.F., Smith M., Quevedo J., Frey B.N. (2020). Bipolar disorder in women: menstrual cycle, perinatal period, and menopause transition. Women’s Ment. Heal..

[b0120] Fredly H., Ersvær E., Kittang A.O., Tsykunova G., Gjertsen B.T., Bruserud Ø. (2013). The combination of valproic acid, all-trans retinoic acid and low-dose cytarabine as disease-stabilizing treatment in acute myeloid leukemia. Clin. Epigenetics.

[b0125] Fushida S., Kinoshita J., Kaji M., Oyama K., Hirono Y., Tsukada T., Fujimura T., Ohta T. (2016). Paclitaxel plus valproic acid versus paclitaxel alone as second-or third-line therapy for advanced gastric cancer: a randomized Phase II trial. Drug Des. Devel. Ther..

[b0130] Gad A.M. (2018). Study on the influence of caffeic acid against sodium valproate–induced nephrotoxicity in rats. J. Biochem. Mol. Toxicol..

[b0135] Galaly S.R., Abdella E.M., Mohammed H.M. (2014). Effects of royal jelly on genotoxicity and nephrotoxicity induced by valproic acid in albino mice. Beni-Suef Univ. J. basic Appl. Sci..

[b0140] Garay F.J.L., Loureiro N.E. (2015). Neuroprotective action of valproic acid accompanied of the modification on the expression of Bcl-2 and activated caspase-3 in the brain of rats submitted to ischemia/reperfusion. Invest. Clin..

[b0145] Gayam V., Mandal A.K., Khalid M., Shrestha B., Garlapati P., Khalid M. (2018). Valproic acid induced acute liver injury resulting in hepatic encephalopathy-a case report and literature review. J. Community Hosp. Intern. Med. Perspect..

[b0150] Georgoff P.E., Nikolian V.C., Bonham T., Pai M.P., Tafatia C., Halaweish I., To K., Watcharotone K., Parameswaran A., Luo R. (2018). Safety and tolerability of intravenous valproic acid in healthy subjects: a phase I dose-escalation trial. Clin. Pharmacokinet..

[b0155] Gerstner T., Buesing D., Longin E., Bendl C., Wenzel D., Scheid B., Goetze G., Macke A., Lippert G., Klostermann W. (2006). Valproic acid induced encephalopathy–19 new cases in Germany from 1994 to 2003–a side effect associated to VPA-therapy not only in young children. Seizure.

[b0160] Gezginci-Oktayoglu S., Turkyilmaz I.B., Ercin M., Yanardag R., Bolkent S. (2016). Vitamin U has a protective effect on valproic acid-induced renal damage due to its anti-oxidant, anti-inflammatory, and anti-fibrotic properties. Protoplasma.

[b0165] Ghodke-Puranik Y., Thorn C.F., Lamba J.K., Leeder J.S., Song W., Birnbaum A.K., Altman R.B., Klein T.E. (2013). Valproic acid pathway: pharmacokinetics and pharmacodynamics. Pharmacogenet. Genomics.

[b0170] Go H.S., Seo J.E., Kim K.C., Han S.M., Kim P., Kang Y.S., Han S.H., Shin C.Y., Ko K.H. (2011). Valproic acid inhibits neural progenitor cell death by activation of NF-κB signaling pathway and up-regulation of Bcl-XL. J. Biomed. Sci..

[b0175] Gopaul S.V., Farrell K., Abbott F.S. (2000). Identification and Characterization ofN-Acetylcysteine Conjugates of Valproic Acid in Humans and Animals. Drug Metab. Dispos..

[b0180] Grillo M.P., Chiellini G., Tonelli M., Benet L.Z. (2001). Effect of α-fluorination of valproic acid on valproyl-S-acyl-CoA formation in vivo in rats. Drug Metab. Dispos..

[b0185] Grosso S., Mostardini R., Piccini B., Balestri P. (2009). Body mass index and serum lipid changes during treatment with valproic acid in children with epilepsy. Ann. Pharmacother..

[b0190] Guenter R., Patel Z., Chen H. (2021). Notch signaling in thyroid cancer. Notch Signal. Embryol. Cancer.

[b0195] Hamed S.A. (2017). The effect of antiepileptic drugs on the kidney function and structure. Expert Rev. Clin. Pharmacol..

[b0200] Handoko K.B., Souverein P.C., Van Staa T.P., Meyboom R.H.B., Leufkens H.G.M., Egberts T.C.G., Van Den Bemt P.M.L.A. (2006). Risk of Aplastic Anemia in Patients Using Antiepileptic Drugs. Epilepsia.

[b0205] Hani A.J., Husain A.M. (2018). Early Treatment of Convulsive Status Epilepticus. Status Epilepticus. Springer.

[b0210] Heidari R., Jafari F., Khodaei F., Shirazi Yeganeh B., Niknahad H. (2018). Mechanism of valproic acid-induced Fanconi syndrome involves mitochondrial dysfunction and oxidative stress in rat kidney. Nephrology.

[b0215] Hommers L. (2020). Mood Stabilizers: Pharmacology and Biochemistry. NeuroPsychopharmacotherapy.

[b0220] Iamsaard S., Sukhorum W., Arun S., Phunchago N., Uabundit N., Boonruangsri P., Namking M. (2017). Valproic acid induces histologic changes and decreases androgen receptor levels of testis and epididymis in rats. Int. J. Reprod. Biomed..

[b0225] Ibrahim I.H., Aboregela A.M., Gouda R.H.E., Eid K.A. (2019). Chronic valproate treatment influences folliculogenesis and reproductive hormones with possible ameliorating role for folic acid in adult albino rats. Acta Histochem..

[b0230] Jagtap S.A. (2020). Older Antiepileptic Drugs. Antiepileptic. Drugs.

[b0235] Jamora D., Lim S., Pan A., Tan L., Tan E. (2007). Valproate-induced Parkinsonism in epilepsy patients. Mov. Disord..

[b0240] Jawed S., Kim B., Ottenhof T., Brown G.M., Werstiuk E.S., Niles L.P. (2007). Human melatonin MT1 receptor induction by valproic acid and its effects in combination with melatonin on MCF-7 breast cancer cell proliferation. Eur. J. Pharmacol..

[b0245] Johannessen C.U., Johannessen S.I. (2003). Valproate: past, present, and future. CNS Drug Rev..

[b0250] Kang S.-H., Seok Y.M., Song M., Lee H.-A., Kurz T., Kim I. (2015). Histone deacetylase inhibition attenuates cardiac hypertrophy and fibrosis through acetylation of mineralocorticoid receptor in spontaneously hypertensive rats. Mol. Pharmacol..

[b0255] Kasiviswanathan R., Longley M.J., Chan S.S.L., Copeland W.C. (2009). Disease Mutations in the Human Mitochondrial DNA Polymerase Thumb Subdomain Impart Severe Defects in Mitochondrial DNA Replication*. J. Biol. Chem..

[b0260] Kassahun K., Hu P., Grillo M.P., Davis M.R., Jin L., Baillie T.A. (1994). Metabolic activation of unsaturated derivatives of valproic acid. Identification of novel glutathione adducts formed through coenzyme A-dependent and-independent processes. Chem. Biol. Interact..

[b0265] Khan S., Ahmad T., Parekh C.V., Trivedi P.P., Kushwaha S., Jena G. (2011). Investigation on sodium valproate induced germ cell damage, oxidative stress and genotoxicity in male Swiss mice. Reprod. Toxicol..

[b0270] Kiang T.K.L., Ho P.C., Anari M.R., Tong V., Abbott F.S., Chang T.K.H. (2006). Contribution of CYP2C9, CYP2A6, and CYP2B6 to valproic acid metabolism in hepatic microsomes from individuals with the CYP2C9* 1/* 1 genotype. Toxicol. Sci..

[b0275] Kim T., Song S., Park Y., Kang S., Seo H. (2019). HDAC inhibition by valproic acid induces neuroprotection and improvement of PD-like behaviors in LRRK2 R1441G transgenic mice. Exp. Neurobiol..

[b0280] Koeck J.A., Hilgarth H., von Ameln-Mayerhofer A., Meyn D., Warlich R., Münstedt A., Horn D., König C. (2021). Clinically Relevant Interactions with Anti-Infectives on Intensive Care Units—A Multicenter Delphi Study. Antibiotics.

[b0285] Komulainen T., Lodge T., Hinttala R., Bolszak M., Pietilä M., Koivunen P., Hakkola J., Poulton J., Morten K.J., Uusimaa J. (2015). Sodium valproate induces mitochondrial respiration dysfunction in HepG2 in vitro cell model. Toxicology.

[b0290] LaBuzetta J.N., Yao J.Z., Bourque D.L., Zivin J. (2010). Adult nonhepatic hyperammonemia: a case report and differential diagnosis. Am. J. Med..

[b0295] Lamparter C., Winn L.M. (2014). Tissue-specific effects of valproic acid on DNA repair genes and apoptosis in postimplantation mouse embryos. Toxicol. Sci..

[b0300] Leng Y., Chuang D.-M. (2006). Endogenous α-synuclein is induced by valproic acid through histone deacetylase inhibition and participates in neuroprotection against glutamate-induced excitotoxicity. J. Neurosci..

[b0305] Leppik I.E., Birnbaum A.K. (2010). Epilepsy in the elderly. Ann. N. Y. Acad. Sci..

[b0310] Levy S.B., Marshall B. (2004). Antibacterial resistance worldwide: causes, challenges and responses. Nat. Med..

[b0315] Li R., Cao S., Fang W., Song Y., Luo X.-T., Wang H., Wang J. (2017). Roles of HDAC2 and HDAC8 in cardiac remodeling in renovascular hypertensive rats and the effects of valproic acid sodium. Pharmacology.

[b0320] Li S., Guo J., Ying Z., Chen S., Yang L., Chen K., Long Q., Qin D., Pei D., Liu X. (2015). Valproic acid-induced hepatotoxicity in Alpers syndrome is associated with mitochondrial permeability transition pore opening-dependent apoptotic sensitivity in an induced pluripotent stem cell model. Hepatology.

[b0325] Li W., Ma L. (2019). Synergistic antitumor activity of oridonin and valproic acid on HL-60 leukemia cells. J. Cell. Biochem..

[b0330] Li H., Zhang Z., Gao C., Wu S., Duan Q., Wu H., Wang C., Shen Q., Yin T. (2019). Combination chemotherapy of valproic acid (VPA) and gemcitabine regulates STAT3/Bmi1 pathway to differentially potentiate the motility of pancreatic cancer cells. Cell Biosci..

[b0335] Lin T., Ren Q., Zuo W., Jia R., Xie L., Lin R., Zhao H., Chen J., Lei Y., Wang P. (2019). Valproic acid exhibits anti-tumor activity selectively against EGFR/ErbB2/ErbB3-coexpressing pancreatic cancer via induction of ErbB family members-targeting microRNAs. J. Exp. Clin. Cancer Res..

[b0340] Liu X.S., Chopp M., Kassis H., Jia L.F., Hozeska-Solgot A., Zhang R.L., Chen C., Cui Y.S., Zhang Z.G. (2012). Valproic acid increases white matter repair and neurogenesis after stroke. Neuroscience.

[b0345] Liu Y., Li S., Zhang Z., Lv Z., Jiang H., Tan X., Liu F. (2017). Effects of valproic acid on sympathetic activity and left ventricularmyocardial remodelling in rats during pressure overload. Turkish J. Med. Sci..

[b0350] Lloyd K.A. (2013). A scientific review: mechanisms of valproate-mediated teratogenesis. Biosci. Horizons Int. J. Student Res..

[b0355] López-Muñoz F., Baumeister A.A., Hawkins M.F., Álamo C. (2012). The role of serendipity in the discovery of the clinical effects of psychotropic drugs: beyond of the myth. Actas españolas Psiquiatr..

[b0360] Löscher W. (2002). Basic pharmacology of valproate: a review after 35 years of clinical use for the treatment of epilepsy. CNS Drugs.

[b0365] Löscher W., Nau H. (1983). Distribution of valproic acid and its metabolites in various brain areas of dogs and rats after acute and prolonged treatment. J. Pharmacol. Exp. Ther..

[b0370] Maneenin C., Burawat J., Maneenin N., Nualkaew S., Arun S., Sampannang A., Iamsaard S. (2018). Antioxidant capacity of momordica charantia extract and its protective effect on testicular damage in valproic acid-induced rats. Int. J Morphol..

[b0375] Mezghani N., Mnif M., Kacem M., Mkaouar-Rebai E., Hadj Salem I., Kallel N., Charfi N., Abid M., Fakhfakh F. (2011). A whole mitochondrial genome screening in a MELAS patient: a novel mitochondrial tRNAVal mutation. Biochem. Biophys. Res. Commun..

[b0380] Michaelis M., Suhan T., Cinatl J., Driever P.H., Cinatl J. (2004). Valproic acid and interferon-α synergistically inhibit neuroblastoma cell growth in vitro and in vivo. Int. J. Oncol..

[b0385] Mishra M.K., Kukal S., Paul P.R., Bora S., Singh A., Kukreti S., Saso L., Muthusamy K., Hasija Y., Kukreti R. (2021). Insights into Structural Modifications of Valproic Acid and Their Pharmacological Profile. Molecules.

[b0390] Moavero R., Pisani L.R., Pisani F., Curatolo P. (2018). Safety and tolerability profile of new antiepileptic drug treatment in children with epilepsy. Expert Opin. Drug Saf..

[b0395] Monti B., Gatta V., Piretti F., Raffaelli S.S., Virgili M., Contestabile A. (2010). Valproic acid is neuroprotective in the rotenone rat model of Parkinson’s disease: involvement of α-synuclein. Neurotox. Res..

[b0400] Muangsab J., Prommeenate P., Chetsawang B., Chonpathompikunlert P., Sukketsiri W., Hutamekalin P. (2019). Protective effect of valproic acid on MPP+-induced neurotoxicity in dopaminergic SH-SY5Y cells through Cdk5/p35/Erk signaling cascade. Trop. J. Pharm. Res..

[b0405] Nelson-DeGrave V.L., Wickenheisser J.K., Cockrell J.E., Wood J.R., Legro R.S., Strauss J.F., McAllister J.M. (2004). Valproate potentiates androgen biosynthesis in human ovarian theca cells. Endocrinology.

[b0410] Nguyen K.V., Sharief F.S., Chan S.S.L., Copeland W.C., Naviaux R.K. (2006). Molecular diagnosis of Alpers syndrome. J. Hepatol..

[b0415] Nicolai J., Aldenkamp A.P., Huizenga J.R., Teune L.K., Brouwer O.F. (2007). Cognitive side effects of valproic acid-induced hyperammonemia in children with epilepsy. J. Clin. Psychopharmacol..

[b0420] Ornoy A. (2009). Valproic acid in pregnancy: how much are we endangering the embryo and fetus?. Reprod. Toxicol..

[b0425] Ornoy A., Becker M., Weinstein-Fudim L., Ergaz Z. (2020). S-adenosine methionine (SAME) and valproic acid (VPA) as epigenetic modulators: Special emphasis on their interactions affecting nervous tissue during pregnancy. Int. J. Mol. Sci..

[b0430] Paradis F., Hales B.F. (2015). Valproic acid induces the hyperacetylation of P53, expression of P53 target genes, and markers of the intrinsic apoptotic pathway in midorganogenesis murine limbs. Birth defects Res. part B Dev. Reprod. Toxicol..

[b0435] Park H.K., Han B.R., Park W.H. (2020). Combination of arsenic trioxide and valproic acid efficiently inhibits growth of lung cancer cells via G2/M-phase arrest and apoptotic cell death. Int. J. Mol. Sci..

[b0440] Peña-Ortega F. (2019). Brain arrhythmias induced by amyloid beta and inflammation: involvement in Alzheimer’s disease and other inflammation-related pathologies. Curr. Alzheimer Res..

[b0445] Phiel C.J., Zhang F., Huang E.Y., Guenther M.G., Lazar M.A., Klein P.S. (2001). Histone deacetylase is a direct target of valproic acid, a potent anticonvulsant, mood stabilizer, and teratogen. J. Biol. Chem..

[b0450] Phillips A., Bullock T., Plant N. (2003). Sodium valproate induces apoptosis in the rat hepatoma cell line, FaO. Toxicology.

[b0455] Rahman M., Nguyen H. (2021).

[b0460] Ren M., Leng Y., Jeong M., Leeds P.R., Chuang D. (2004). Valproic acid reduces brain damage induced by transient focal cerebral ischemia in rats: potential roles of histone deacetylase inhibition and heat shock protein induction. J. Neurochem..

[b0465] Rocca A., Minucci S., Tosti G., Croci D., Contegno F., Ballarini M., Nolè F., Munzone E., Salmaggi A., Goldhirsch A. (2009). A phase I-II study of the histone deacetylase inhibitor valproic acid plus chemoimmunotherapy in patients with advanced melanoma. Br. J. Cancer.

[b0470] Savran M., Ascı H., Armagan İ., Erzurumlu Y., Azırak S., Kaya Ozer M., Bilgic S., Korkmaz D.T. (2020). Thymoquinone could be protective against valproic acid-induced testicular toxicity by antioxidant and anti-inflammatory mechanisms. Andrologia.

[b0475] Schäfer M., Brandl E.J. (2020). Mood stabilizers: valproate. NeuroPsychopharmacotherapy.

[b0480] Schmitt B., Martin F., Critelli H., Molinari L., Jenni O.G. (2009). Effects of valproic acid on sleep in children with epilepsy. Epilepsia.

[b0485] Scholz B., Schulte J.S., Hamer S., Himmler K., Pluteanu F., Seidl M.D., Stein J., Wardelmann E., Hammer E., Völker U. (2019). HDAC (histone deacetylase) inhibitor valproic acid attenuates atrial remodeling and delays the onset of atrial fibrillation in mice. Circ. Arrhythmia Electrophysiol..

[b0490] Sharma S., Sarathlal K.C., Taliyan R. (2019). Epigenetics in neurodegenerative diseases: the role of histone deacetylases. CNS Neurol. Disord. Targets (Formerly Curr. Drug Targets-CNS Neurol. Disord..

[b0495] Shi X., Liu Y., Zhang D., Xiao D. (2019). Valproic acid attenuates sepsis-induced myocardial dysfunction in rats by accelerating autophagy through the PTEN/AKT/mTOR pathway. Life Sci..

[b0500] Shirsath N., Rathos M., Chaudhari U., Sivaramakrishnan H., Joshi K. (2013). Potentiation of anticancer effect of valproic acid, an antiepileptic agent with histone deacetylase inhibitory activity, by the cyclin-dependent kinase inhibitor P276–00 in human non-small-cell lung cancer cell lines. Lung Cancer.

[b0505] Silva M.R., Correia A.O., Dos Santos G.C.A., Parente L.L.T., de Siqueira K.P., Lima D.G.S., Moura J.A., da Silva Ribeiro A.E., Costa R.O., Lucetti D.L. (2018). Neuroprotective effects of valproic acid on brain ischemia are related to its HDAC and GSK3 inhibitions. Pharmacol. Biochem. Behav..

[b0510] Singh D., Gupta S., Verma I., Morsy M.A., Nair A.B., Ahmed A.-S.-F. (2021). Hidden pharmacological activities of valproic acid: A new insight. Biomed. Pharmacother..

[b0515] Spartalis E., Athanasiadis D.I., Chrysikos D., Spartalis M., Boutzios G., Schizas D., Garmpis N., Damaskos C., Paschou S.A., Ioannidis A. (2019). Histone deacetylase inhibitors and anaplastic thyroid carcinoma. Anticancer Res..

[b0520] Su J.M., Li X.-N., Thompson P., Ou C.-N., Ingle A.M., Russell H., Lau C.C., Adamson P.C., Blaney S.M. (2011). Phase 1 study of valproic acid in pediatric patients with refractory solid or CNS tumors: a children’s oncology group report. Clin. Cancer Res..

[b0525] Sun J., Piao J., Li N., Yang Y., Kim K., Lin Z. (2020). Valproic acid targets HDAC1/2 and HDAC1/PTEN/Akt signalling to inhibit cell proliferation via the induction of autophagy in gastric cancer. FEBS J..

[b0530] Sveberg Røste L., Taubøll E., Isojärvi J.I.T., Pakarinen A.J., Huhtaniemi I.T., Knip M., Gjerstad L. (2002). Effects of chronic valproate treatment on reproductive endocrine hormones in female and male Wistar rats. Reprod. Toxicol..

[b0535] Tan L., Yu J.-T., Sun Y.-P., Ou J.-R., Song J.-H., Yu Y. (2010). The influence of cytochrome oxidase CYP2A6, CYP2B6, and CYP2C9 polymorphisms on the plasma concentrations of valproic acid in epileptic patients. Clin. Neurol. Neurosurg..

[b0540] Terranova-Barberio M., Roca M.S., Zotti A.I., Leone A., Bruzzese F., Vitagliano C., Scogliamiglio G., Russo D., D’Angelo G., Franco R. (2016). Valproic acid potentiates the anticancer activity of capecitabine in vitro and in vivo in breast cancer models via induction of thymidine phosphorylase expression. Oncotarget.

[b0545] Tian S., Lei I., Gao W., Liu L., Guo Y., Creech J., Herron T.J., Xian S., Ma P.X., Chen Y.E. (2019). HDAC inhibitor valproic acid protects heart function through Foxm1 pathway after acute myocardial infarction. EBioMedicine.

[b0550] Trombley T.A., Capstick R.A., Lindsley C.W. (2019). DARK Classics in Chemical Neuroscience: Gamma-Hydroxybutyrate (GHB). ACS Chem. Neurosci..

[b0555] Tsai J., Wu T., Leung H., Desudchit T., Tiamkao S., Lim K., Dash A. (2018). Perampanel, an AMPA receptor antagonist: from clinical research to practice in clinical settings. Acta Neurol. Scand..

[b0560] Vajda F. (2012). Dose issues in antiepileptic therapy. J. Clin. Neurosci..

[b0565] Van den Berg R.J., Kok P., Voskuyl R.A. (1993). Valproate and sodium currents in cultured hippocampal neurons. Exp. brain Res..

[b0570] Verrotti A., Agostinelli S., Parisi P., Chiarelli F., Coppola G. (2011). Nonalcoholic fatty liver disease in adolescents receiving valproic acid. Epilepsy Behav..

[b0575] Verrotti A., Mencaroni E., Cofini M., Castagnino M., Leo A., Russo E., Belcastro V. (2016). Valproic acid metabolism and its consequences on sexual functions. Curr. Drug Metab..

[b0580] Verrotti A., Iapadre G., Di Donato G., Di Francesco L., Zagaroli L., Matricardi S., Belcastro V., Iezzi M.L. (2019). Pharmacokinetic considerations for anti-epileptic drugs in children. Expert Opin. Drug Metab. Toxicol..

[b0585] Wang Z., Tsai L.-K., Munasinghe J., Leng Y., Fessler E.B., Chibane F., Leeds P., Chuang D.-M. (2012). Chronic valproate treatment enhances postischemic angiogenesis and promotes functional recovery in a rat model of ischemic stroke. Stroke.

[b0590] Wei M., Mao S., Lu G., Li L., Lan X., Huang Z., Chen Y., Zhao M., Zhao Y., Xia Q. (2018). Valproic acid sensitizes metformin-resistant human renal cell carcinoma cells by upregulating H3 acetylation and EMT reversal. BMC Cancer.

[b0595] Xie Z., Ago Y., Okada N., Tachibana M. (2018). Valproic acid attenuates immunosuppressive function of myeloid-derived suppressor cells. J. Pharmacol. Sci..

[b0600] Ximenes J.C.M., Neves K.R.T., Leal L.K.A.M., Do Carmo M.R.S., Brito G.A.deC., Naffah-Mazzacoratti M.da.G., Cavalheiro É.A., Viana G.S.de.B. (2015). Valproic acid neuroprotection in the 6-OHDA model of Parkinson’s disease is possibly related to its anti-inflammatory and HDAC inhibitory properties. J. Neurodegener Dis..

[b0605] Xuan A., Long D., Li J., Ji W., Hong L., Zhang M., Zhang W. (2012). Neuroprotective effects of valproic acid following transient global ischemia in rats. Life Sci..

[b0610] Yan L., Yang K., Wang S., Xie Y., Zhang L., Tian X. (2021). PXR-mediated expression of FABP4 promotes valproate-induced lipid accumulation in HepG2 cells. Toxicol. Lett..

[b0615] Zhang C., Zhu J., Zhang J., Li H., Zhao Z., Liao Y., Wang X., Su J., Sang S., Yuan X. (2014). Neuroprotective and anti-apoptotic effects of valproic acid on adult rat cerebral cortex through ERK and Akt signaling pathway at acute phase of traumatic brain injury. Brain Res..

[b0620] Zhang C., Liu S., Yuan X., Hu Z., Li H., Wu M., Yuan J., Zhao Z., Su J., Wang X. (2016). Valproic acid promotes human glioma U87 cells apoptosis and inhibits glycogen synthase kinase-3β through ERK/Akt signaling. Cell. Physiol. Biochem..

[b0625] Zhang Z., Zhang R., Hao C., Pei X., Li J., Wang L. (2020). GANT61 and valproic acid synergistically inhibited multiple myeloma cell proliferation via Hedgehog signaling pathway. Med. Sci. Monit. Int. Med. J. Exp. Clin. Res..

[b0630] Zheng Z., Wu Y., Li Z., Ye L., Lu Q., Zhou Y., Yuan Y., Jiang T., Xie L., Liu Y. (2020). Valproic acid affects neuronal fate and microglial function via enhancing autophagic flux in mice after traumatic brain injury. J. Neurochem..

[b0635] Zobdeh F., Ben Kraiem A., Attwood M.M., Chubarev V.N., Tarasov V.V., Schiöth H.B., Mwinyi J. (2021). Pharmacological treatment of migraine: Drug classes, mechanisms of action, clinical trials and new treatments. Br. J. Pharmacol..

